# Piperine functions as a tumor suppressor for human ovarian tumor growth via activation of JNK/p38 MAPK-mediated intrinsic apoptotic pathway

**DOI:** 10.1042/BSR20180503

**Published:** 2018-05-28

**Authors:** Lihui Si, Ruiqi Yang, Ruixin Lin, Shuli Yang

**Affiliations:** 1 Department of Obstetrics and Gynecology, the Second Hospital of Jilin University, Changchun 130041, P.R. China; 2 Department of General Surgery, the Second Hospital of Jilin University, Changchun 130041, P.R. China

**Keywords:** Apoptosis, Caspase, JNK, Mitochondria, p38 MARK, Piperine

## Abstract

Piperine, a kind of natural alkaloid found in the fruit of black (*Piper nigrum* Linn) and long (*Piper longum* Linn), has shown antitumor activities toward various cancer cell lines. However, the antitumor effects of Piperine on ovarian cancer and the underlying mechanism are not fully elucidated. Our result showed that Piperine reduced the cell viability of A2780 cells in a concentration and time-dependent manner, but has not any effect on normal ovarian cells. Flow cytometric analysis revealed that Piperine suppressed cells proliferation via induction of apoptosis, which was followed by release of mitochondrial cytochrome *c* to cytosol, activation of caspase-3 and -9, as well as cleaved PARP. Moreover, Western blot results confirmed that Piperine (8, 16, and 20 μM) decreased phosphorylation of JNK and p38 MAPK in A2780 cells. In addition, caspase-3 inhibitor (Z-DEVD-FMK), caspase-9 inhibitor (Z-LEDH-FMK), JNK-inhibitor (SP600125), or p38 MAPK inhibitor (SB203580) could abate the apoptosis induced by Piperine (20 μM) treatment, while caspase-8 inhibitor (Z-IETD- FMK) exhibited no inhibitory effect on the induction of apoptosis in A2780 cells. These results provide the first evidence for the anticancer potential of Piperine in ovarian cancer cells, partially via JNK/p38 MAPK-mediated intrinsic apoptotic pathway.

## Introduction

Ovarian cancer remains the most lethal gynecologic malignancies among women [1]. Due to lack of early typical symptoms and effective screening strategies, approximately 70% of the patients have been diagnosed at late stage [2]. Although traditional surgical resection and cisplatin/paclitaxel-based chemotherapy have been currently introduced to advanced ovarian cancer treatment [3], it is found that the 5-year survival rate of patients is less than 40% [4,5]. The treatments for ovarian cancer are typically unsuccessful and there are still increasing chemoresistance of ovarian cancer and high rates of metastasis and recurrence [6,7]. Multidrug resistance of the cancer cells to chemotherapy is a formidable challenge in the field of cancer chemotherapy. Therefore, there is a highly urgent need for searching novel effective and less cytotoxic agents for the treatment of ovarian cancer patients.

Recently, many kinds of bioactive phytochemicals have provided a great contribution to the development of the new therapies for chemoresistant ovarian cancers due to their structural diversity [8–12]. They demonstrate minimal toxicity and adverse side effects, thereby making them optimal alternative medicine to conventional cytotoxic chemotherapy [13]. Phytochemicals derived from spices, including peppers, are recognized to be an important resource for developing potential antitumor agents [14]. Piperine, a major alkaloidal constituent presented in black (*Piper nigrum* Linn) and long (*Piper longum* Linn), has been accepted extensively as one of the most common spices used in food and folk medicine worldwide [15,16]. A load of evidence from previous studies demonstrated that it possesses a wide range of pharmacological actions, such as anticonvulsant [17], antimicrobial [18], antioxidant [19], neuroprotective [20], anti-inflammatory, and antiarthritic activities [21,22]. Importantly, it is also known to prevent tumor development in various cancers, including breast cancer, lung cancer, prostate cancer, gastric cancer, rectal cancer, and so on [21,23–26]. Additionally, earlier studies showed that colchicine administration could prevent and delay the development of aflatoxin and CCl_4_-induced cancer in rats without significant side effects [27]. However, the antitumor effect of Piperine on ovarian carcinoma is still not elucidated. Therefore, our goal of the present study is to investigate whether Piperine has inhibitory effect on the growth of ovarian cancer cells *in vitro*, as well as further explore the underlying molecular mechanism.

## Materials and methods

### Materials and chemicals

Piperine (>95% purity), 3-[4,5-dimethylthiazol-2-yl]-2,5-diphenyltetrazolium bromide (MTT), dimethylsulfoxide (DMSO), 4,6-diamidino-2-phenylindole (DAPI), rhodamine 123 (Rho-123), and protease inhibitors were purchased from Sigma Chemical (St. Louis, MO). Dulbecco modified Eagle medium (DMEM), fetal bovine serum (FBS), penicillin, Akt inhibitor LY294002, p38 MAPK inhibitor SB203580, JNK1/2 inhibitor SP600125, and ERK1/2 inhibitor PD98059 were from Invitrogen (Grand Island, NY, U.S.A.). Annexin V-FITC Apoptosis Detection kit was from Invitrogen (Carlsbad, CA, U.S.A). BCA Protein Assay Kit was from Pierce (Rockford, IL, U.S.A). Primary antibodies for β-actin, cytosolic cytochrome *c*, phosphorylated p38 MAPK (p-p38 MAPK) and p38 MAPK, phosphorylated JNK (p-JNK), and cleaved PARP were purchased from Santa Cruz Biotechnology (Santa Cruz, CA, U.S.A). Caspase-3, caspase-8, and caspase-9 colorimetric assay kits are from Kengen Inc, (Beijing, China). All other chemicals and reagents were of analytical grade.

### Cell lines and culture conditions

Human ovarian cancer cell line A2780 was purchased from American Type Culture Collection (ATCC) and human ovarian epithelial cell line OSE was obtained from the Institute of Cell and Biochemistry Research of Chinese Academy of Science (Shanghai, China). They are all cultured in DMEM medium supplemented with 10% FBS, 100 U/ml penicillin, and 100 μg/ml streptomycin at 37°C with 5% CO_2_ in a humidified incubator.

### Cell treatment

Piperine was freshly prepared by dissolving in DMSO before any treatment. The prepared DMSO stock solution of Piperine was used to make final defined concentrations (4–20 μM) in culture medium. Controls were prepared by preparing 0.07% solution of DMSO in culture media in all experiments. The cancer cells were exposed to ZVAD-FMK (50 μM) for 60 min to inhibit the caspase activity before treatment with Piperine (20 μM) for 48 h. The cancer cells were also treated in the presence or absence of JNK-inhibitor (20 μM) / MAPK inhibitor p38 for 60 min followed by treatment of Piperine (20 μM) for 48 h before evaluating phosphorylation levels of JNK and p38 MAPK.

### MTT assay

The effect of Piperine on the viability of cells was detected by MTT assay. Briefly, A2780 cells and OSE cells at the logarithmic phase were seeded in a 96-well plate at a concentration of 1 × 10^4^/well in a 96-well plate overnight. After being treated with Piperine for 12, 24, 48, and 72 h at the concentration of 20 μM, or with indicated concentrations of Piperine (0, 4, 8, 16, or 20 μM) for 48 h, 20 μl of MTT solution (5 mg/ml in PBS) was added to each well and incubated for another 4 h at 37°C. Then, the MTT solution was removed and DMSO (100 μl) was added to each well to dissolve the MTT formazan. The absorbance was measured at 540 nm with a microplate reader.

### Observation of morphological changes

The logarithmic phase of A2780 cells were seeded into six-well plates in the density of 4 × 10^4^/well and cultured overnight. After treatment with different concentrations of Piperine (8, 16, or 20 μM) for 48 h, the cellular morpholocgy was observed with the Axiovert200 inverted microscope (Carl Zeiss, Oberkochen, Germany).

### DAPI staining assay

In brief, after ovarian cancer cells were seeded into 24-well plates at approximately 4 × 10^4^ cells/well and treated with Piperine at 0, 8, 16, or 20 μM for 48 h. Cells in each were well fixed with 3.7% formaldehyde, followed by staining with DAPI. The cells were then washed with PBS and photographed (excitation maxima 358 nm; emission maxima 461 nm) by fluorescence microscope (OLYMPUS, Essex, U.K.).

### Flow cytometry analysis

The extent of apoptosis was evaluated by flow cytometry using Annexin V-FITC Apoptosis Detection kit as per the manufacturer’s instructions. Briefly, treated cancer cells were harvested and washed, and then the cells were incubated with Annexin V-FITC and PI for 10 min in the dark. Finally, a total of 10,000 cells were subsequently collected and analyzed by FACS/Calibur flow cytometer (Becton Dickin-son, Franklin Lakes, NJ, U.S.A).

### Analysis of caspase activities

Caspase-3, caspase-8, and caspase-9 activities were performed using a colorimetric assay kit according to the manufacturer’s instructions. Briefly, after cells were incubated with Piperine at indicated concentrations for 48 h, the lysis buffer was added to the cell on ice for 10 min and centrifuged at 12000×***g*** for 10 min at 4°C. The supernatants were collected in a 96-well flat bottom microplate to assess caspase activity. Each well consisted of 50 μl of cell lysate and 50 μl of reaction buffer 3, 8, or 9. Then, for each reaction, 5 μl of caspase-3, caspase-8, or caspase-9 colorimetric substrate (LEHD-pNA) was added to each well and then incubated at 37°C for 1 h. Finally, absorbance was measured on a microplate reader at an absorbance of 405 nm.

### Determination of mitochondrial membrane potential (ΔΨm)

The collapse of the inner ΔΨm was measured using the fluorescent dye, Rh123. In the method, the cells were subjected to seeding in a six-well plate and were treated with varying concentrations of Piperine for 48 h. Then, the cells were washed with PBS and stained with Rh123 (10 mM) for 30 min in the dark at 37°C. The mean fluorescence intensity (MFI) of cancer cells was determined using flow cytometric analysis.

### Western blot analysis

To examine the cytochrome *c* release from mitochondria in untreated or Piperine treated cells, cytosolic extracts were prepared using digitonin-permeabilization technique [28]. Briefly, cells treated with or without Piperine were washed twice with PBS and lysed in ice-cold lysis buffer containing 70 mM Tris and 250 mM sucrose at pH 7.0. After 20 min of incubation on ice, cells were immediately centrifuged at 12000×***g*** for 5 min at 4°C and the supernatant was stored for analysis of cytochrome *c* in cytosol, and the pellets were dissolved in lysis buffer for analysis of cytochrome *c* in mitochondria.

For whole cell lysates, cells were lysed in ice-cold lysis buffer containing 1% protease inhibitors and the protein contents of the lysates were determined using a BCA Protein Assay Kit. Each equal amount of protein samples (10 μg) were separated by 10% SDS-PAGE, transferred onto nitrocellulose membrane and then blocked with 5% nonfat milk for 2 h at room temperature. After blocking, the membranes were probed with specific primary antibody overnight at 4°C, followed by incubation with corresponding HRP-conjugated secondary antibodies for 1 h. Then, specific protein bands were visualized using the ECL kit according to the manufacturer’s instructions. Densitometric analysis of the blots was performed using the QuantityOne software (Bio-Rad).

### Statistical analysis

The results were expressed as mean ± SD (standard deviation) of triplicate experiments. Statistical significances were evaluated by the two-tailed Student’s *t*-test and one-way analysis of variance (one-way ANOVA). *P<0.05 and **P<0.01 were considered as the significant difference.

## Results

### Piperine inhibited cell viability of A2780 cells

**Figure 1:**
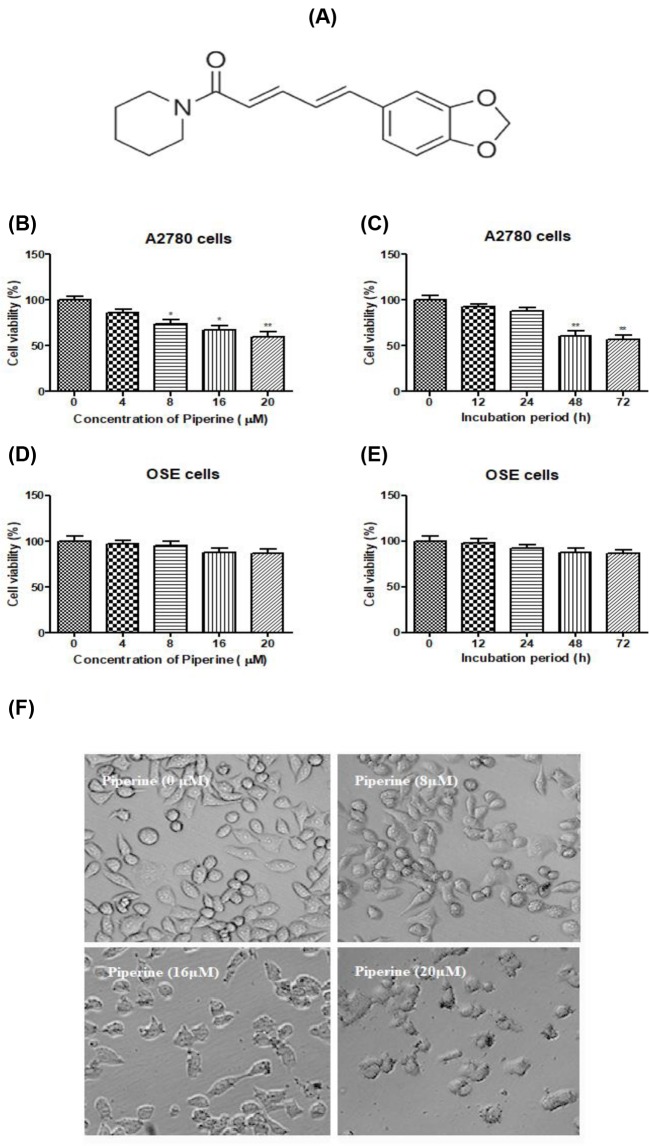
Effect of Piperine on cell viability of human ovarian cancer A2780 cells and human ovarian epithelial OSE cells. (**A**) The chemical structure of Piperine. (**B**) Effect of Piperine (0, 4, 8, 16, or 20 μM) on cell viability of ovarian cancer cell line A2780 at 48 h. (**C**) Effect of Piperine (20 μM) on cell viability of ovarian cancer cell line A2780 at 12, 24, 48, and 72 h. (**D**) Effect of Piperine (0, 4, 8, 16, or 20 μM) on cell viability of normal ovarian cell line OSE at 48 h. (**E**) Effect of Piperine (20 μM) on cell viability of normal ovarian cell line OSE at 12, 24, 48, and 72 h. (**F**) Effect of Piperine (8, 16, or 20 μM) treatment for 48 h on the morphological changes of ovarian cancer cell line A2780 and the representative images were photographed with inverted contrast microscopy at 200× magnification. Data shown are mean ± SD of three similar experiments. *P<0.05 and **P<0.01 as compared with the control group.

The chemical structure of Piperine is shown in Figure 1A. For determining the antiproliferation effects of Piperine on ovarian cancer cells, cell viability was detected by MTT assay. The results in Figure 1B,C showed that Piperine decreased the survival rate of A2780 cells in a dose- and time-dependent manner, which was significantly different from control cells, especially at 8, 16, or 20 μM and after 48 h treatment period (*P*<0.05 or *P*<0.01). No obvious cell viability loss was observed on OSE cells (Figure 1D,E; *P*>0.05). Moreover, morphologic change of A2780 cells was observed upon treatment with Piperine for 48 h (Figure 1F). The untreated ovarian cancer cells displayed normal abundant cytoplasmic vacuoles after 48 h, but the cells treated with Piperine started to shrink, became round, and finally detached from the culture flask compared with the untreated cells. These data suggested that Piperine inhibited the proliferation of A2780 cells and leading to cell death.

### Piperine causes apoptosis of A2780 cells

**Figure 2:**
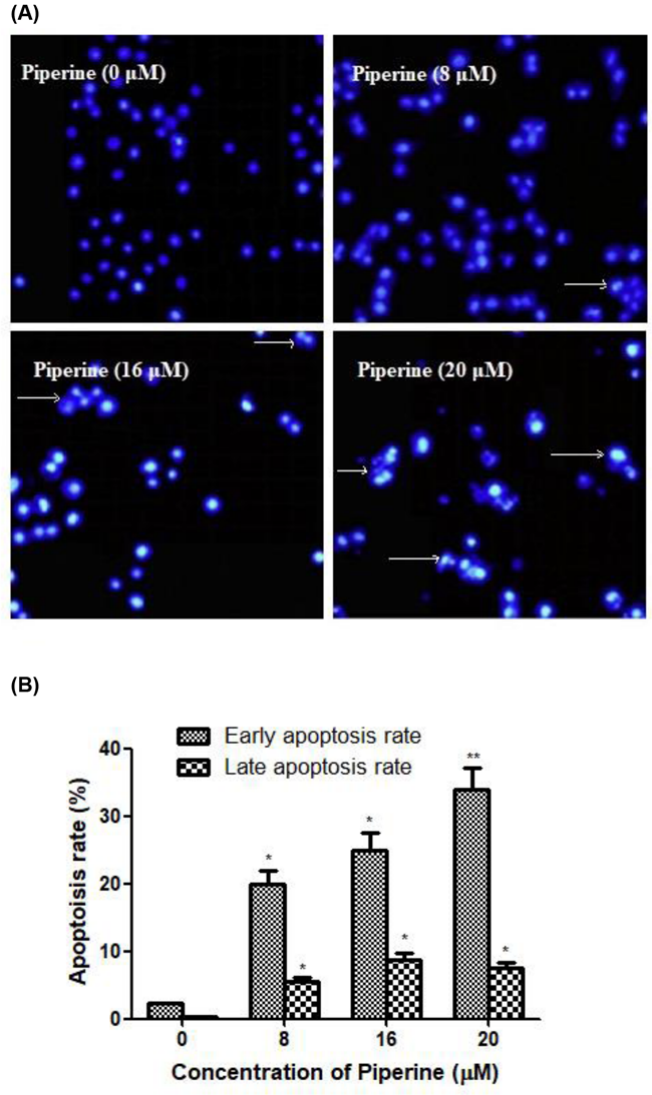
Effect of Piperine on apoptosis of human ovarian cancer A2780 cells. (**A**) The morphology of the cell nucleus in ovarian cancer cell line A2780 treated with Piperine (0, 8, 16, or 20 μM) was observed after DAPI staining by fluorescence microscopy at 100× magnification. Nuclear condensation and apoptotic bodies were indicated by the arrows. (**B**) Effect of Piperine (0, 8, 16, or 20 μM) on the percentages of apoptotic cells of ovarian cancer cell line A2780 (including early stage and late stage) by quantification of FACS analysis. Data shown are mean ± SD of three similar experiments. *P<0.05 and **P<0.01 as compared with the control group.

To further assess whether the growth inhibitory effects of Piperine on A2780 cells were related to apoptosis, the ovarian cancer cells treated with or without Piperine were stained with DAPI and photographed by a fluorescence microscopy (Figure 2A). Treatment with the Piperine for 48 h led to a significant increase in the number of cells with nuclear chromatin condensation and fragmented nuclei, while the control cells displayed normal and intact nuclei. It suggested that Piperine might be able to induce ovarian cancer cell apoptosis.

Supporting this result, the percentages of apoptotic cells were analyzed using flow cytometry (Figure 2B). After treatment with different concentrations of Piperine (8, 16, or 20 μM) for 48 h, the numbers of early and late apoptotic cells were significantly increased as compared with respective control (untreated) counterparts.

### Piperine induces the apoptosis via disruption of ΔΨm and activation of caspases

**Figure 3:**
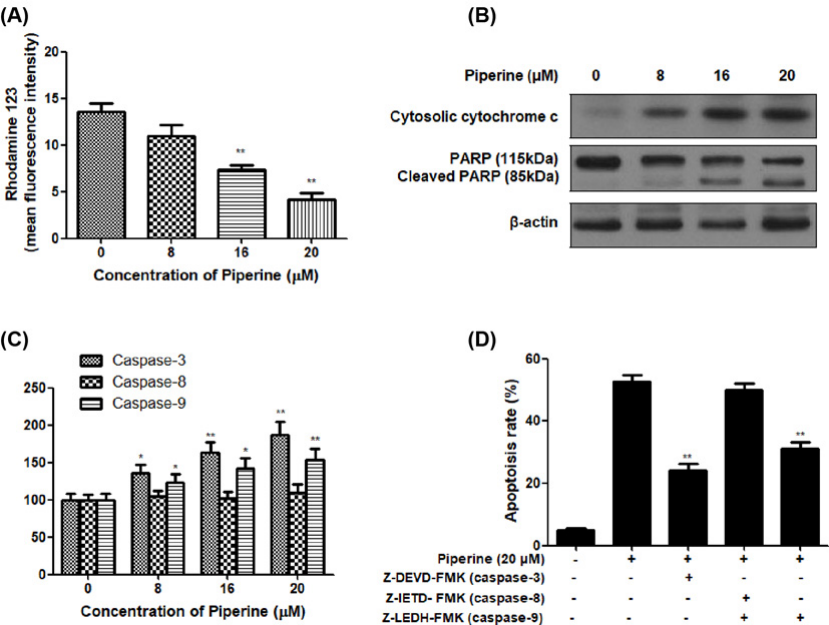
Involvement of mitochondria dependent pathway in Piperine-induced apoptosis in human ovarian cancer A2780 cells. (**A**) Effect of Piperine (0, 8, 16, or 20 μM) on the mitochondrial membrane potential (ΔΨm) of ovarian cancer cell line A2780. (**B**) Effect of Piperine (0, 8, 16, or 20 μM) on the cytosolic cytochrome *c*, PARP, and cleaved PARP protein expression of ovarian cancer cell line A2780. (**C**) Effect of Piperine (0, 8, 16, or 20 μM) on caspase-3, caspase-8, and caspase-9 activities of ovarian cancer cell line A2780. (**D**) Effect of specific caspase inhibitors Z-DEVD-FMK (caspase-3), Z-IETD-FMK (caspase-8), or Z-LEDH-FMK (caspase-9) on apoptosis of ovarian cancer cell line A2780 induced by Piperine (20 μM). Data shown are mean ± SD of three similar experiments. *P<0.05 and **P<0.01 as compared with the control group.

Further to analyze the effect of Piperine on mitochondria of A2780 cells, the cells treated with Piperine (8, 16, or 20 μM) for 48 h were exposed to fluorescent dye Rh123. The results of the experiment suggested a significant decrease in MFI of Rh123 in A2780 cells treated with Piperine, highly significant results were seen in cells treated with 16 and 20 μM of Piperine (Figure 3A), indicating that Piperine treatment induced destruction of ΔΨm in A2780 cells.

We next examined the change of cytochrome *c* and caspase activity in A2780 cells after exposure to Piperine (8, 16, or 20 μM) for 48 h. As shown in Figure 3B, Piperine (8, 16, or 20 μM) treatment for 48 h dose-dependently resulted in increased levels of cytochrome *c* release from mitochondria. The released cytochrome *c* can further activate caspase-9 and caspase-3. As anticipated, the activities of caspase-3 and caspase-9 increased greatly along with the rising concentration of Piperine, whereas caspase-8 remains unchanged (Figure 3C). In order to make certain which pathway of apoptosis execution involved, A2780 cell cells were pretreated with specific caspase inhibitors Z-DEVD-FMK (caspase-3), Z-IETD-FMK (caspase-8), or Z-LEDH-FMK (caspase-9) for 1 h before 20 μM of Piperine treatment, and then cells were harvested and subjected to flow cytometric analysis to detect apoptotic cells rate. It was clearly that Piperine-induced apoptosis was greatly reduced by Z-DEVD and Z-LEDH, but not Z-IETD (Figure 3D). In addition, PARP, a preferential substrate for caspase-3, caused a PARP cleavage in a dose-dependent manner in A2780 cells upon exposure to Piperine (Figure 3B), which was evidenced by the presence of the characteristic 85 kDa fragments and a concomitant attenuation of the 115 kDa full-length PARP protein. Together, our data demonstrated that the involvement of an intrinsic/mitochondria dependent pathway in the apoptosis of A2780 cells following Piperine treatment.

### Piperine induces apoptotic cell death via modulation of JNK and p38 MAPK signaling pathways

**Figure 4:**
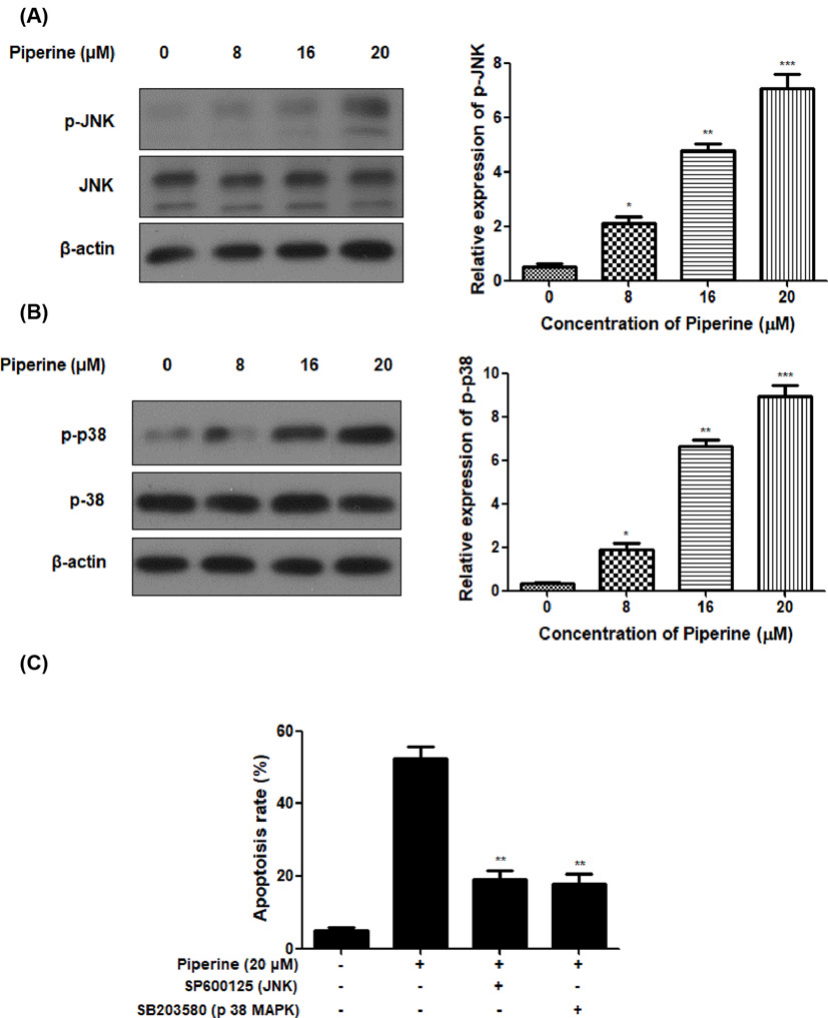
Piperine induces apoptotic cell death via modulation of JNK and p38 MAPK signaling pathways in human ovarian cancer A2780 cells. (**A**) Effect of Piperine (0, 8, 16, or 20 μM) on phosphorylation of JNK of ovarian cancer cell line A2780. (**B**) Effect of Piperine (0, 8, 16, or 20 μM) on p38 MAPK of ovarian cancer cell line A2780. (**C**) Effect of JNK-inhibitor or p38 MAPK inhibitor on apoptosis of ovarian cancer cell line A2780 induced by Piperine (20 μM). Data shown are mean ± SD of three similar experiments. *P<0.05, **P<0.01 and ***P<0.01 as compared to the control group.

In this experiment, JNK and p38 MAPK were also examined in A2780 cells after Piperine treatment by Western blot analysis. Piperine treatment at the concentration of 8, 16, or 20 μM resulted in a visible decrease in phosphorylation of JNK and p38 MAPK protein expression in A2780 cells (Figure 4A,B). To further evaluate the relevance of JNK and p38 MAPK pathways in controlling the apoptotic cell death by Piperine, 20 μM of specific inhibitor of SP600125 (JNK) or SB203580 (p38 MAPK) was added to A2780 cells for 60 min prior to treatment of Piperine (20 μM) for 48 h. The percentage of apoptosis determined by flow cytometry in Figure 4C showed that JNK-inhibitor or p38 MAPK inhibitor significantly reduced the apoptosis rate of A2780 cells as compared with those in cells treated Piperine (20 μM) alone. This experimental outcomes suggested Piperine-mediated apoptosis in A2780 cells was associated with the activation of JNK and p38 MAPK.

## Discussion

Ovarian cancer is a deadly gynecological disease and still faces the challenging recurrence of drug resistant clones following chemotherapy [29]. In spite of improvement in postsurgical chemotherapy with paclitaxel plus carboplatin, the relapse rate is still high at over 50%. Natural products derived from herbal medicines drawn more and more attention for cancer treatment, due to severe side effects and drug resistance of the traditional chemotherapeutics [30,31]. Therefore, in the present study, we explored cytotoxic effects of Piperine against ovarian cancer cells, and endeavored to analyze the mechanisms underlining the observed effects.

In the present study, we observed that Piperine showed dose- and time-dependent antiproliferation activities against human ovarian A2780 cells, but not human ovarian epithelial OSE cells. It was also found that ovarian cancer cells treated with Piperine become round and detached from the culture flask. All data suggested that Piperine may prevent the proliferation of ovarian cancer cells.

Apoptosis or programmed cell death plays very important roles in controlling physiological cell growth and tissue homeostasis [32,33]. Any uncontrolled cell proliferation may thus result from the accumulation of apoptosis-related genes, leading to the loss of programmed cell death control [34]. Therefore, induction of apoptosis became the major target of most of anticancer agents. The present data in DAPI staining assay indicated that ovarian cancer A2780 cells treated with Piperine displayed specific apoptotic morphological changes. Additionally, flow cytometric data further indicated that the percentage of early and late apoptotic cells dramatically increased in A2780 cells following Piperine treatment. All these data suggested that Piperine induced apoptosis in ovarian cancer cells. To better understand the underlying mechanisms, we further investigate the molecular changes during this process. We first evaluated the influence of Piperine treatment on the mitochondrial membrane potential of A2780 cells using fluorescent dye Rh123. A decrease in the fluorescence of Rh123 indicates a loss of ΔΨm, which is reported to decrease in cells undergoing apoptosis [35,36]. As expected, ΔΨm showed a dose-dependent decrease in Piperine-treated A2780 cells at the concentration of 8, 16, or 20 μM. Upon the damage of mitochondrial transmembrane potential, the cytochrome *c* would be released from the mitochondria into the cytoplasm, leading to activation of caspase-3 and caspase-9, thus initiating the mitochondrial pathway of apoptosis [37,38]. After 48 h of coculture A2780 cells with Piperine, Western blot analysis showed that cytochrome *c* protein expression became more evident along with increasing concentration of Piperine. Coincidentally, we found that treatment of A2780 cells with Piperine increased the caspase-3 and caspase-9 activities following 48-h treatment, whereas the level of caspase-8 remains unchanged in A2780 cells under identical conditions, indicating that the apoptotic cell death in A2780 cells induced by Piperine may be due to the activation of intrinsic apoptotic pathways. In addition, pretreatment of cells with the specific caspase-3 inhibitor (Z-DEVD-FMK), caspase-8 inhibitor (Z-IETD- FMK), or caspase-9 inhibitor (Z-LEDH-FMK) for 1 h prior to Piperine (20 μM) treatment, and then apoptotic cells rate was examined in A2780 cells. Both caspase-3 inhibitor and caspase-9 inhibitor could block the apoptosis induced by Piperine at the concentration of 20 μM, while caspase-8 inhibitor didn’t have any inhibitory effect on the induction of apoptosis in A2780 cells. In agreement with activation of caspase-3, Piperine treatment triggered a dose-dependent proteolytic cleavage of PARP (a classical substrate of caspase-3), as evidenced by the accumulation of the characteristic 85 kDa fragments and a concomitant disappearance of the 115 kDa full-length PARP protein, further confirming the role of caspase-3 activation in the Piperine-induced apoptosis in ovarian cancer A2780 cells [39]. Presently, two dominant caspase-dependent apoptotic signal pathways have been reported. In general, one is the mitochondrial-mediated apoptotic (intrinsic) pathway where caspase-9 is engaged by accompanying with disruption of mitochondrial potential [40], and the other is the death receptor-mediated apoptotic (extrinsic) pathway in which tumor necrosis factor family activates upstream caspase-8 [41,42]. Therefore, the activation of caspase-3 and caspase-9 indicates the involvement of the mitochondria-initiated intrinsic apoptotic pathway in Piperine-induced apoptosis in ovarian cancer A2780 cells, rather than the death receptor-dependent extrinsic apoptotic pathway.

To gain more insight into the mechanisms of Piperine-induced apoptosis in ovarian cancer A2780 cells, we analyzed two important apoptotic signaling proteins: JNK and p38 MAPK by Western blot analysis. It is well known that JNK/p38 MAPK pathway is critical for cancer development and progression by regulating several physiological processes, including apoptosis, proliferation, survival, and differentiation [43,44]. Outcomes of our study revealed that the addition of Piperine resulted in increased phosphorylation of JNK and p38 MAPK in A2780 cells. Further, in order to evaluate the status of JNK and p38 MAPK in Piperine-mediated apoptosis, the A2780 cells were exposed to JNK-inhibitor or p38 MAPK inhibitor for 60 min followed by treatment of Piperine (20 μM) for 48 h. The apoptosis rate of A2780 cells induced by Piperine was dramatically palliated by exposure to Piperine. In conclusion, we have demonstrated, for the first time, that Piperine could induce cell death through the JNK/p38 MAPK-mediated intrinsic apoptotic pathway in ovarian cancer cells. This finding suggested that Piperine may be used as an anticancer agent for future treatment of ovarian cancer.

## Abbreviations

DAPI: 4,6-diamidino-2-phenylindole
DMEM: Dulbecco modified Eagle medium
DMSO: dimethylsulfoxide
FACS: fluorescence activated cell sorting
FBS: fetal bovine serum
HRP: horseradish peroxidase
JNK: c-Jun N-terminal kinase
MAPK: mitogen-activated protein kinase
MFI: mean fluorescence intensity
MTT: 3-[4,5-dimethylthiazol-2-yl]-2,5-diphenyltetrazolium bromide
OSE: ovarian epithelial cell
PI: propidium iodide
